# Tonic excitation by astrocytic GABA causes neuropathic pain by augmenting neuronal activity and glucose metabolism

**DOI:** 10.1038/s12276-024-01232-z

**Published:** 2024-05-17

**Authors:** Yeon Ha Ju, Jongwook Cho, Ji-Young Park, Hyunjin Kim, Eun-Bin Hong, Ki Duk Park, C. Justin Lee, Euiheon Chung, Hyoung-Ihl Kim, Min-Ho Nam

**Affiliations:** 1 Brain Science Institute, Korea Institute of Science and Technology (KIST), Seoul, 02792 Republic of Korea; 2 Department of Biomedical Science and Engineering, Gwangju Institute of Science and Technology (GIST), Gwangju, 61005 Republic of Korea; 3 Center for Cognition and Sociality, Institute for Basic Science, Daejeon, 34126 Republic of Korea; 4 Department of Neurosurgery, Presbyterian Medical Center, Jeonju, 54987 Republic of Korea; 5 Department of KHU-KIST Convergence Science and Technology, Kyung Hee University, Seoul, 02447 Republic of Korea

**Keywords:** Astrocyte, Neurotransmitters, Neuropathic pain

## Abstract

Neuropathic pain is a debilitating condition caused by the hyperexcitability of spinal dorsal horn neurons and is often characterized by allodynia. Although neuron-independent mechanisms of hyperexcitability have been investigated, the contribution of astrocyte-neuron interactions remains unclear. Here, we show evidence of reactive astrocytes and their excessive GABA release in the spinal dorsal horn, which paradoxically leads to the tonic excitation of neighboring neurons in a neuropathic pain model. Using multiple electrophysiological methods, we demonstrated that neuronal hyperexcitability is attributed to both increased astrocytic GABA synthesis via monoamine oxidase B (MAOB) and the depolarized reversal potential of GABA-mediated currents (E_GABA_) via the downregulation of the neuronal K^+^/Cl^−^ cotransporter KCC2. Furthermore, longitudinal 2-deoxy-2-[^18^F]-fluoro-D-glucose microPET imaging demonstrated increased regional glucose metabolism in the ipsilateral dorsal horn, reflecting neuronal hyperexcitability. Importantly, inhibiting MAOB restored the entire astrocytic GABA-mediated cascade and abrogated the increased glucose metabolism and mechanical allodynia. Overall, astrocytic GABA-mediated tonic excitation is critical for neuronal hyperexcitability, leading to mechanical allodynia and neuropathic pain.

## Astrocytic GABA cascade: key to neuropathic pain mechanism

Neuropathic pain can greatly affect a person’s life by causing disability and financial strain. The cause of this pain is complex and many factors have been studied to understand it. In this research, it was found that neuropathic pain comes from overactive and overly sensitive neurons in the spinal cord after a nerve injury. This overactivity can be caused by a paradoxical tonic excitation by astrocytic GABA, which is mediated by an impaired homeostasis of chloride ion at the spinal dorsal horn. The research was done on rats and involved surgery, drug use, and various tests. The results showed that blocking the production of astrocytic GABA can decrease the abnormally high neuronal activity and metabolism, ultimately reducing mechanical allodynia. The researchers concluded that adjusting the astrocytic GABA production could be a potential treatment for neuropathic pain.

This summary was initially drafted using artificial intelligence, then revised and fact-checked by the author.

## Introduction

Neuropathic pain is a condition that can severely impact a person’s quality of life and is distinct from acute nociceptive pain, as it does not resolve during the healing process and can cause individual disability and pose socioeconomic burdens^1,2^. Neuropathic pain can develop from injury to the somatosensory nervous system when deleterious changes occur in the injured nerve and along pain pathways. The prevalence of neuropathic pain is 6.9–10% in the general population^3^. Neuropathic pain is clinically characterized by mechanical allodynia, hyperalgesia, and burning pain, which are not commonly observed in nociceptive pain^2,4^. Despite various therapeutic approaches, neuropathic pain often remains medically intractable. Further research is therefore required to understand its pathophysiology and the mechanisms that can contribute to the control of this intractable state.

The development of neuropathic pain is multifactorial, and various mechanisms have been investigated to elucidate its pathophysiology. It is widely accepted that neuropathic pain originates from hyperexcitability and sensitization in the peripheral and central nervous systems following nerve injury. Hyperexcitability can be induced by the abnormal attenuation of GABA-mediated inhibitory signals, resulting in a decreased response to incoming nociceptive stimuli in the spinal dorsal horn under physiological conditions^5^. Recent reports have demonstrated that inhibitory signals are disrupted by a reduction in the functional expression of the potassium-chloride cotransporter (KCC2) in the spinal dorsal horn^6,7^. Specifically, reduced KCC2 expression has been shown to disrupt neuronal chloride ion (Cl^−^) homeostasis, reducing the ability of GABA to decrease the cell’s excitability and, ultimately contributing to the development of neuropathic pain^5,8^. Notably, the decrease in KCC2 expression has been attributed to brain-derived neurotrophic factor (BDNF) released from reactive microglia^9,10^, a main component of neuroinflammation with reactive astrocytes.

In the past two decades, the non-neuronal cells such as microglia and astrocytes have been identified as key contributors to chronic pain in addition to neurons^11^. In particular, astrocytes are well known to be involved in reactive gliosis in response to neuroinflammation following nerve injury, which is characterized by the upregulation of glial fibrillary acidic protein (GFAP) and/or morphological changes, including hypertrophy, proliferation, and modifications of glial networks^12^. Glial modulators released from astrocytes, including cytokines, chemokines, proteases, glutamate, ATP, D-serine, and prostaglandin E2, contribute to maintaining chronic pain and generating neuropathic symptoms^11,13,14^. In addition, reactive astrocytes release excessive GABA into the extrasynaptic space in response to acute and chronic neuroinflammation following nervous system injury^15^. However, the role of astrocytic GABA and its interaction with disrupted KCC2 homeostasis are not fully understood in the context of neuropathic pain.

In this study, we investigated the pathological implications of astrocytic GABA in the spinal dorsal horn in a neuropathic pain model induced by L5 spinal nerve ligation (SNL). Specifically, we tested the hypothesis that SNL leads to abnormal tonic excitation of spinal dorsal horn neurons, which is attributable to a pronounced shift in the functional role of astrocytic GABA from inhibitory to excitatory due to decreased KCC2 expression. To locate and monitor the aberrant tonic excitation of neuronal activity in the spinal cord, we utilized micropositron emission tomography (microPET) with an ^18^F-fluorodeoxyglucose (^18^F-FDG) tracer, which allows functional imaging of glucose metabolic activity. Based on our previous studies showing that MAOB inhibitors selectively reduce astrocytic GABA levels and improve behavior under other disease conditions^15,16^, we further investigated the potential beneficial effect of a MAOB inhibitor on reducing the severity of neuropathic pain. In addition, we validated the potential of FDG-microPET for monitoring therapeutic efficacy.

## Materials and methods

### Rats

All experimental animal procedures were conducted in accordance with the National Institutes of Health guidelines and approved by the Institutional Animal Ethical Committee, Korea Institute of Science and Technology (KIST; Seoul, Korea, Approval Number KIST-2020-177) and Gwangju Institute of Science and Technology (GIST; Gwangju, Korea, Approval Number GIST-2021-043). Eight-week-old male SD rats were used for the experiments. All rats were co-housed at the animal facility at a temperature of 21 ± 2 °C, a relative humidity of 50 ± 15%, and a 12 h light/dark cycle (lights on and off at 8 am and 8 pm). All rats were provided with food and water ad libitum and were randomly allocated to each experimental group. All experiments were performed with age-matched controls, and different sets of animals were used for each experiment.

### Surgical procedures

Rats underwent left L5-SNL surgery as described by Kim and Chung^17^. Under deep anesthesia with 2% isoflurane, a midline incision was made from the L4 to the S2 level. After removing the subcutaneous tissue, the transverse process was carefully removed to identify the L4 and L5 spinal nerves. The L5 spinal nerve was carefully isolated without damage and tightly ligated with 6–0 silk thread (AILEE Co., Busan, Korea). The same surgical procedure was performed in the sham-operated group with the exception of nerve ligation. After confirming complete hemostasis, the wound was sutured.

### Intrathecal drug administration

After SNL surgery, the intrathecal catheter was implanted using the procedure described by Yaksh and Ruby^18^. Briefly, rats were anesthetized with 2% isoflurane. The rats were shaved on the back of the neck and fixed with ear bars on a rodent stereotactic frame. The skin over the nape of the neck was incised approximately 1 cm. After detaching and retracting the muscle of the external occipital crest, the atlanto-occipital membrane was incised with a needle. The catheter was made from stretchable and flexible polyethylene tubing (I.D. 0.28 mm, O.D. 0.061 mm, 427401; BD Intramedic, Franklin Lakes, NJ) attached to the osmotic pump via larger glued-on tubing (I.D. 0.76 mm, O.D. 1.22 mm #BB31785-V/4; Scientific Commodities Inc., Lake Havasu City, AZ). A catheter was slowly inserted into the spinal subarachnoid space until it reached the lumbar enlargement. Then, the exit end of the catheter was connected to an osmotic pump (ALZET Model 2ML2, Cupertino, CA, USA). A pocket was formed by spreading apart the subcutaneous connective tissues between the scapulae. The pump was inserted into the pocket. The incision site in the skin was sutured. The pump continuously delivered KDS2010 (10 mg/kg/day, pumping rate 5 μL/h) for 14 days. Catheter-implanted rats showing no apparent dysfunction were used for experiments.

### Von Frey test

Paw withdrawal thresholds to mechanical stimuli were measured by von Frey filaments (Touch-Test Sensory Evaluator; North Coast Medical Inc., Morgan Hill, CA). The test was conducted on the rats prior to surgery (baseline) and on Days 1, 3, 7, 11, and 15 after SNL surgery. Each rat was placed on a metallic mesh floor covered with a transparent acryl cage and allowed to habituate for 20 min. A series of von Frey filaments (3.61, 3.84, 4.08, 4.31, 4.56, 4.74, 4.93, 5.18) were applied to the plantar surface of the hind paw using the up-down method, and the 50% paw withdrawal threshold was calculated as described by Chaplan^19^. Rats with a low mechanical threshold presurgery (less than 10 g) and rats without mechanical hypersensitivity at 3 days following SNL surgery were excluded from the study. A withdrawal threshold of 15 g was used as the cutoff value. After SNL surgery, rats with thresholds ≤4 g were considered to have developed neuropathic pain.

### Immunohistochemistry

After completing the last von Frey test, the rats were perfused with 0.9% saline followed by 4% paraformaldehyde (PFA), and the L4/L5 segments of the spinal cord were removed. The removed spinal cord segments were post-fixed with 4% paraformaldehyde for 12 h and cryoprotected with 30% sucrose. The lumbar region of the spinal cord was transversely sectioned at a thickness of 40 µm.

For immunofluorescence staining, tissues were incubated for 1.5 h in blocking solution (0.3% Triton-X, 1% goat serum in PBS) at room temperature and then incubated with a mixture of primary antibodies in blocking solution at 4 °C on a shaker overnight. After washing in PBS 3 times, the sections were incubated with the corresponding fluorescent secondary antibodies for 2 h and then washed with PBS 3 times. Finally, the sections were mounted on glass slides with gel-mounting medium. The primary antibodies used for fluorescent immunostaining were as follows: chicken anti-GFAP (1:500; Millipore ab5541), rabbit anti-IBA1 (1:300; Proteintech 10904-1-AP), mouse anti-NeuN (1:1000; Millipore MAB377), guinea pig anti-GABA (1:200; Millipore ab175), rabbit anti-MAOB (1:300; Abcam ab137778), and rabbit anti-KCC2 (1:500; Millipore 07-432). Fluorescent secondary antibodies were purchased from Invitrogen or Jackson ImmunoResearch and used at a 1:500 dilution. A series of fluorescence images were obtained with an A1 Nikon confocal microscope, and 3-μm-thick Z-stack images were processed for further analysis using NIS-Elements (Nikon) software, the ImageJ program (NIH), and Imaris 9 (Bitplane). Any alterations in brightness or contrast were equally applied to the entire image set. The specificity of the primary antibody and immunoreaction was confirmed by omitting the primary antibodies or changing the fluorescent probes of the secondary antibodies.

### PET/CT image acquisition and preprocessing

PET and CT images were acquired to measure changes in regional glucose metabolism in the L5 segment of the spinal cord. The animals underwent three scanning sessions: the first scan was performed prior to the SNL surgery (baseline), and the second and third scans were performed on Days 4 and 14 after the SNL surgery (PO 4 and PO 14). Rats were deprived of food for 12 h prior to PET image scanning to maintain consistent blood glucose levels. 2-Deoxy-2-[18 F]-fluoro-D-glucose (1.5 mCi/kg) was injected via the tail vein under isoflurane anesthesia (1.5%). After a 1-h uptake period, the animals were anesthetized with 1.5% isoflurane and placed in the prone position on a microPET/CT scanner bed (Siemens Medical Solutions, TN, USA). Then, we performed a static PET scan for 25 min and a computed tomography scan for 10 min. During the scanning procedures, body temperature and respiratory and heart rates were monitored (BioVet, m2m Imaging Corp., Cleveland, OH, USA). After the CT scan, the rats were returned to their home cage. The PET and CT images were reconstructed using the 3D OSEM/MAP iterative algorithm and the filtered back projection algorithm, respectively.

Images were processed and analyzed with the AFNI package (National Institutes of Health, MD, USA). After confirming the superimposition of the CT and PET images, these images were spatially co-registered to the L5 MRI template of the Sprague–Dawley rat and resliced with a voxel size of 0.2 × 0.2 × 0.2 mm. Then, PET images of the L5 segment of the spinal cord were cropped and normalized using an intensity-scaling approach. Images were spatially smoothed with an isotropic Gaussian kernel with a 0.6 mm full width at half maximum.

### Statistical analysis of PET images

PET images were statistically analyzed with a group-level linear mixed-effects model (3D-LME in the AFNI). Preoperative (baseline) and postoperative (PO 4 and PO 14) images were compared. The significance threshold for the statistical maps was *p* < 0.01, and the *p*-value was corrected for multiple comparisons with 3dClustSim in AFNI (α = 0.05, *p* < 0.01, *k* < 28). The statistical maps were overlaid on the L5 MRI template to show areas with substantial activity changes. Region of interest (ROI) analysis was performed to measure changes in regional glucose metabolism after KDS2010 administration.

### Preparation of acute spinal cord slices for electrophysiology

Rats were anesthetized with halothane, and the spinal cords and L3-L5 dorsal and ventral horns of the rats were dissected. The spinal cords were sectioned in ice-cold slicing solution (sucrose, 234 mM; KCl, 2.5 mM; MgSO_4_, 104 mM; NaH_2_PO_4_, 1.25 mM; NaHCO_3_, 24 mM; CaCl_2_-2H_2_O, 0.5 mM; and glucose, 11 mM). Horizontal slices (350 mm thick) were prepared with a vibrating-knife microtome DTK-1000 N (Dosaka, Japan). For stabilization, spinal cord slices were incubated at room temperature for at least 1 h in a solution containing NaCl (124 mM), KCl (3 mM), MgSO_4_ (6.5 mM), NaH_2_PO_4_ (1.25 mM), NaHCO_3_ (26 mM), CaCl_2_-2H_2_O (1 mM), and glucose (10 mM) and simultaneously equilibrated with 95% O_2_/5% CO_2_ at 25 °C.

### Tonic GABA recording

Spinal cord slices were transferred to a recording chamber that was continuously perfused with ACSF solution (flow rate, 2 ml/min). Whole-cell recordings were made from dorsal horn neurons. The holding potential was −70 mV. Pipettes (seal resistance 6–8 MΩ) were filled with internal solution [135 mM CsCl, 4 mM NaCl, 0.5 mM CaCl_2_, 10 mM HEPES, 5 mM EGTA, 2 mM Mg–adenosine triphosphate, 0.5 mM Na2–guanosine triphosphate, and 10 mM QX-314, pH adjusted to 7.2 with CsOH (osmolarity, 278 to 285 mOsm)]. The baseline current was stabilized with d-AP5 (50 μM) and 6-cyano-7-nitroquinoxaline-2,3-dione (20 μM) before measuring the tonic current. Electrical signals were digitized and sampled at 50 μs intervals with Digidata 1440 A and a MultiClamp 700B amplifier (Molecular Devices) using pCLAMP10.2 software. The data were filtered at 2 kHz. The tonic GABA current amplitude was measured by the baseline shift after bicuculline (50 μM) administration using the Clampfit program. Tonic current was measured from baseline to bicuculline treatment. The frequency and amplitude of spontaneous inhibitory post-synaptic currents before bicuculline administration were detected and measured by MiniAnalysis (Synaptosoft).

### sAP firing recording

The cell-attached configuration (seal resistance = 100~1000 MΩ) was used to record spontaneous action potential (sAP) firing. The patch pipettes were filled with internal solution [135 mM KCl, 4 mM NaCl, 0.5 mM CaCl_2_, 10 mM HEPES, 5 mM EGTA, 2 mM Mg–adenosine triphosphate, 0.5 mM Na2–guanosine triphosphate, and 10 mM QX-314, pH adjusted to 7.2 with CsOH (osmolarity, 278–285 mOsm)], and the holding potential was 0 mV. Action potential firings were detected by the presence of a downward and upward deflection in the current trace. After the baseline currents were recorded for more than 5 min, the drugs were applied in a bath containing L655,708 (10 μM) or GABA (10 μM) and bicuculine (50 μM).

### Gramicidin-perforated patch clamp for estimating E_GABA_

To record the reversal potential of GABA-mediated current (E_GABA_), we used the Cl^−^-impermeable gramicidin-perforated patch clamp method. Briefly, the glass pipette solution contained (in mM) 140 K gluconate, 5 EGTA, and 10 HEPES (pH 7.4). Gramicidin was freshly dissolved in DMSO and then diluted into the pipette solution (50 µg/mL). Neurons were voltage clamped at −70 mV, and the currents elicited by puff application of GABA (1 mM) were recorded at membrane potentials at 5 mV steps, ranging from −100 mV to −45 mV, in the presence of 50 µM APV, 20 µM CNQX, 1 µM strychnine, and 1 µM tetrodotoxin. The E_GABA_ was determined by using linear regression to calculate the best fit line for the voltage dependence of chloride-mediated currents and then determining the intercept of the current-voltage line with the abscissa. The input resistance was monitored, and the recording was abandoned if the resistance changed >15%. All signals were recorded with an amplifier (MultiClamp700B; Axon Instruments Inc., Union City, CA), filtered at 1−2 kHz, digitized at 10 kHz, and stored for offline analysis.

### Quantification and statistical analysis

Statistical analyses were performed using Prism 9 (GraphPad Software, Inc.). Differences between two different groups were analyzed using two-tailed Student’s unpaired *t*-tests. For comparisons among multiple groups, either one-way analysis of variance (ANOVA) with Tukey’s multiple comparison test or two-way ANOVA with Tukey’s multiple comparison test was used. To assess the statistical significance of changes in a group induced by a certain intervention, the two-tailed Student’s paired *t*-test or repeated-measures two-way ANOVA with Bonferroni’s multiple comparison test was used. We examined the normality of the distribution for each dataset. In instances where the data did not follow a normal distribution, we conducted suitable nonparametric tests, such as the Mann–Whitney test, Friedman test, or Kruskal‒Wallis ANOVA. To compare two or more groups, we also tested whether the variances were significantly different across the groups. If the variances were different, we applied appropriate corrections to the statistical tests. *P* < 0.05 was considered to indicate statistical significance. The significance level is represented by asterisks (*P < 0.05, **P < 0.01, ***P < 0.001; ns not significant). Unless otherwise specified, all the data are presented as the mean ± SEM. No statistical method was used to predetermine the sample size. Sample sizes were determined empirically based on our previous experiences or a review of similar experiments in the literature. The number of animals used in each experiment is described in the corresponding figure legends or on each graph. All experiments were repeated at least three times to ensure accuracy. The experimental groups were balanced in terms of animal age, sex, and weight. The animals were genotyped before the experiments. All the animals were caged together and treated in the same way. Prior to drug administration, the animals were randomly and evenly allocated to each experimental group. The data from the animal experiments were collected by two independent investigators. However, the investigators were not blinded to the outcome assessments.

## Results

### SNL induces astrocyte reactivity in the ipsilateral spinal dorsal horn

**Fig. 1:**
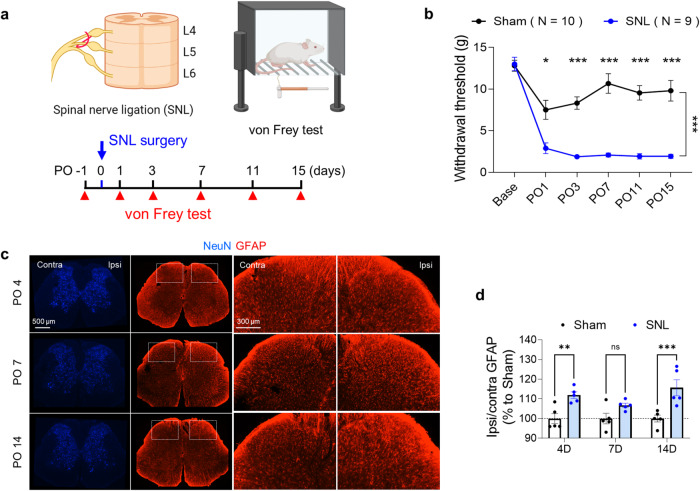
SNL induces reactive gliosis in the ipsilateral spinal dorsal horn. **a** Schematic diagram and experimental timeline of SNL surgery and the von Frey test. **b** Mechanical withdrawal thresholds assessed by the von Frey test in sham- and SNL-operated rats on PO1, 3, 7, 11, and 15 (repeated-measures two-way ANOVA with Bonferroni’s multiple comparisons test, interaction, F (5, 85) = 11.71, *p* < 0.0001; time, F (3.414, 58.04) = 39.49, *p* < 0.0001; group, F (1.17) = 61.60, *p* < 0.0001; *N* = 10 and 9 rats for the Sham and SNL groups, respectively). **c** Immunohistochemistry (NeuN and GFAP) of the dorsal horn of the spinal cord L5 segment in SNL-operated rats at PO4, 7, and 14. **d** Quantification of the GFAP intensity of the ipsilateral dorsal horn in sham- and SNL-operated rats at PO4, 7, and 14 (two-way ANOVA with Sidak’s multiple comparison test, Time F (2, 24) = 1.628, *p* = 0.2172). Group F (1, 24) = 31.76, *p* < 0.0001, (*N* = 5 for each group). The error bars represent the means ± SEMs. *p < 0.05, **p < 0.01, ***p < 0.001, ns non-significant. For detailed statistical information, see also Supplementary Table 1.

To validate the L5-SNL model as a neuropathic pain model, we performed the von Frey test at baseline before surgery and from postoperative (PO) Days 1 to 15 (Fig. 1a) to assess the decrease in the hind limb withdrawal threshold. The SNL model rats showed significant tactile allodynia from PO1 throughout the entire observational period, defined as a 50% response threshold below 3 g of force^20^. In contrast, sham-operated rats did not exhibit any response to light touch (Fig. 1b). All of the SNL-operated rats (9/9) successfully exhibited tactile allodynia within three days, with two-thirds (6/9) even showing signs as early as just one day after the operation.

Since the spinal dorsal horn is the site for integrating peripheral sensory information and transforming touch into pain in a neuropathic pain state, leading to mechanical allodynia^21,22^, we investigated the reactivity of astrocytes in the spinal dorsal horn following the L5-SNL operation. We performed immunohistochemistry to examine GFAP-positive signals in the spinal dorsal horn of L5 segments in the SNL model (Fig. 1c). Consistent with previous studies^23,24^, the intensity of GFAP was significantly greater in the ipsilateral L5 spinal dorsal horn than in the contralateral horn (Fig. 1d). We also observed a moderate increase in GFAP intensity in the ipsilateral ventral horn of SNL-operated rats, indicating the presence of reactive astrogliosis (Supplementary Fig. 1). These findings indicate that reactive astrogliosis is induced in the spinal dorsal horn by the SNL operation.

### Reactive astrocytes excessively release GABA in the dorsal horn

**Fig. 2:**
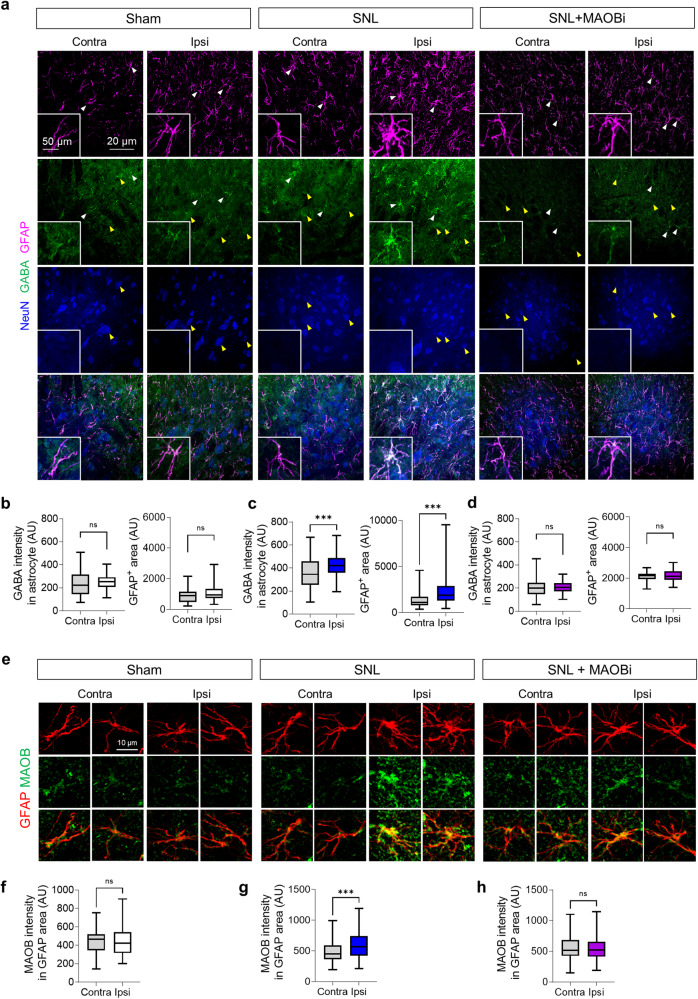
SNL-induced reactive astrocytes exhibit high levels of GABA and MAOB. **a** Immunohistochemistry (GFAP, GABA, and NeuN) of the ipsilateral and contralateral dorsal horns of the L5 segment of the spinal cord in the sham, SNL, and SNL + MAOBi groups (*n* = 4 rats for each group). **b**–**d** Quantification of the intensity of GABA in the GFAP area (sham, two-tailed unpaired *t*-test with Welch’s correction, *p* = 0.3170; SNL, two-tailed unpaired *t*-test with Welch’s correction, *p* < 0.001; SNL + MAOBi, two-tailed unpaired *t*-test with Welch’s correction, *p* = 0.5318) and the area of GFAP (sham, two-tailed unpaired *t*-test, *p* = 0.0853; SNL, two-tailed unpaired *t*-test with Welch’s correction, *p* < 0.001; SNL + MAOBi, two-tailed unpaired *t*-test with Welch’s correction, *p* = 0.1428). **e** Immunohistochemistry (GFAP and MAOB) of the ipsilateral and contralateral dorsal horns of the L5 segment of the spinal cord in the sham, SNL, and SNL + MAOBi groups (n = 4 rats for each group). **f**–**h** Quantification of the intensity of MAOB in the GFAP area (sham, two-tailed unpaired *t*-test, *p* = 0.5461; SNL, two-tailed unpaired *t*-test with Welch’s correction, *p* < 0.001; SNL + MAOBi, two-tailed unpaired *t*-test, *p* = 0.8984). The error bars represent the means ± SEMs. *p < 0.05, **p < 0.01, ***p < 0.001, ns, non-significant. For detailed statistical information, see also Supplementary Table 1.

Accumulating lines of evidence indicate that reactive astrocytes can excessively synthesize and release the inhibitory transmitter GABA in various brain regions^16,25,26^, which, in turn, can decrease neuronal activity and glucose metabolism^15,27^. Thus, we investigated whether reactive astrocytes in the spinal cord also synthesize excessive GABA in a neuropathic pain model. We performed immunohistochemistry on spinal cord tissues from SNL-operated rats with antibodies against GFAP and GABA. We observed GFAP-stained morphologically hypertrophied astrocytes in the ipsilateral dorsal horn, indicative of their reactivity, and a significant increase in astrocytic GABA levels compared to those on the contralateral side (Fig. 2a–d). Furthermore, we examined the expression of monoamine oxidase B (MAOB), a mitochondrial enzyme that is known to be critical for astrocytic GABA synthesis^28^, using immunohistochemistry. We found that the reactive astrocytes in the ipsilateral dorsal horn of the SNL-operated rats showed increased MAOB immunoreactivity (Fig. 2e–h). In contrast, there were no changes in neuronal GABA or MAOB expression following the SNL operation (Supplementary Fig. 2a, b). Additionally, we observed a significant increase in GABA and MAOB immunoreactivity in astrocytes in the ventral horn (Supplementary Fig. 3). To determine whether MAOB is involved in aberrant astrocytic GABA synthesis in the SNL model, we administered a recently developed reversible MAOB inhibitor (MAOBi; 60 mM)^29^ directly to the rat spinal cord through an osmotic pump at an infusion rate of 5 μL/h (10 mg/kg/day), as previously described^18^. We found that pharmacological inhibition of MAOB significantly reduced the expression of astrocytic MAOB and GABA in the ipsilateral dorsal horn (Fig. 2d, h). Taken together, these findings indicate that reactive astrocytes aberrantly synthesize GABA through the enzymatic action of MAOB in neuropathic pain model rats.

**Fig. 3:**
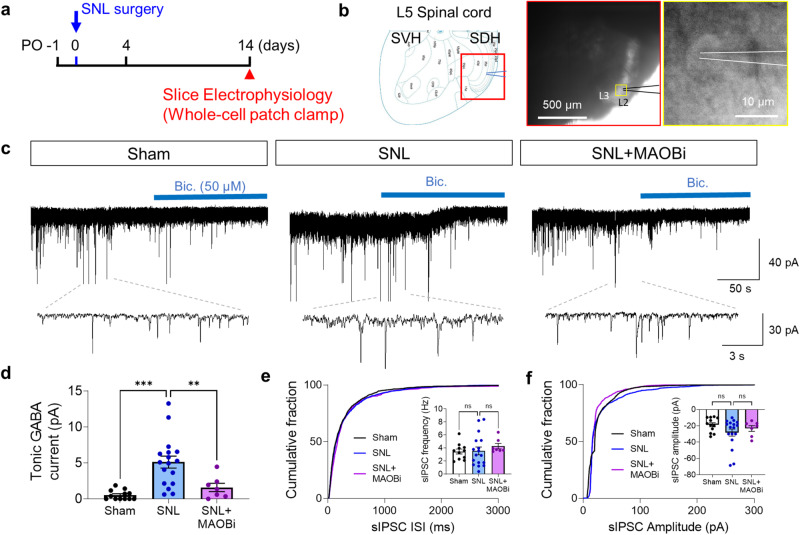
Reactive astrocytes release aberrant GABA in the spinal dorsal horn in the SNL model. **a** Experimental timeline of tonic GABA recording from spinal dorsal horn neurons at the L5 segment. **b** Left, spinal dorsal horn (SDH) as the ROI. Right, representative image of a whole-cell patch clamp in SDH layer II. **c** Representative traces of tonic GABA currents and sIPSCs recorded from SDH neurons in the sham, SNL, and SNL+MAOBi groups (*n* = 3–5 rats for each group). **d** Quantification of tonic GABA currents (pA) in the sham, SNL, and SNL+MAOBi groups (Brown-Forsythe one-way ANOVA with Dunnett T3 multiple comparison test, F (2.000, 23.36) = 19.24, *p* < 0.001), Sham (*n* = 7), SNL (*n* = 17), and SNL + MAOBi (*n* = 7) groups. **e** Quantification of the sIPSC frequency in the sham, SNL, and SNL + MAOBi groups (one-way ANOVA with Tukey’s multiple comparison test, F (2, 32) = 2.966, *p* = 0.6388). Sham (*n* = 7), SNL (*n* = 17), and SNL + MAOBi (*n* = 7) groups. **f** Quantification of the sIPSC amplitude in the sham, SNL, and SNL+MAOBi groups (one-way ANOVA with Tukey’s multiple comparison test, F (2, 33) = 1.698, *p* = 0.1987). Sham (*n* = 7), SNL (*n* = 17), and SNL + MAOBi (*n* = 7) groups. The error bars represent the means ± SEMs. *p < 0.05, **p < 0.01, ***p < 0.001, ns, non-significant. For detailed statistical information, see also Supplementary Table 1.

Next, to investigate the effect of GABA released by astrocytes on neighboring neurons, we performed ex vivo whole-cell patch clamp recordings from spinal dorsal horn layer II and III neurons of sham-operated and SNL-operated rats and recorded the tonic GABA current (Fig. 3a, b). We observed a significantly greater bicuculline (GABA_A_ receptor antagonist)-sensitive tonic GABA current in the SNL-operated rats than in the sham-operated rats (Fig. 3c, d). Furthermore, consistent with our previous findings in the brain^27^, we found that the increase in tonic GABA current was significantly reduced to a normal level by preincubation of acute spinal cord slices with MAOBi (100 nM; Fig. 3c, d). We found no significant differences in the frequency or amplitude of spontaneous inhibitory post-synaptic currents (sIPSCs) across the groups (Fig. 3e, f). Additionally, neither SNL nor MAOBi treatment significantly altered the strychnine (glycine receptor antagonist)-sensitive tonic glycine current (Supplementary Fig. 2c, d). These findings indicate that excessive MAOB-dependent astrocytic GABA tonically binds to GABA_A_ receptors on neighboring neurons in SNL-operated neuropathic pain model rats.

### Reduced KCC2 expression depolarizes E_GABA_

**Fig. 4:**
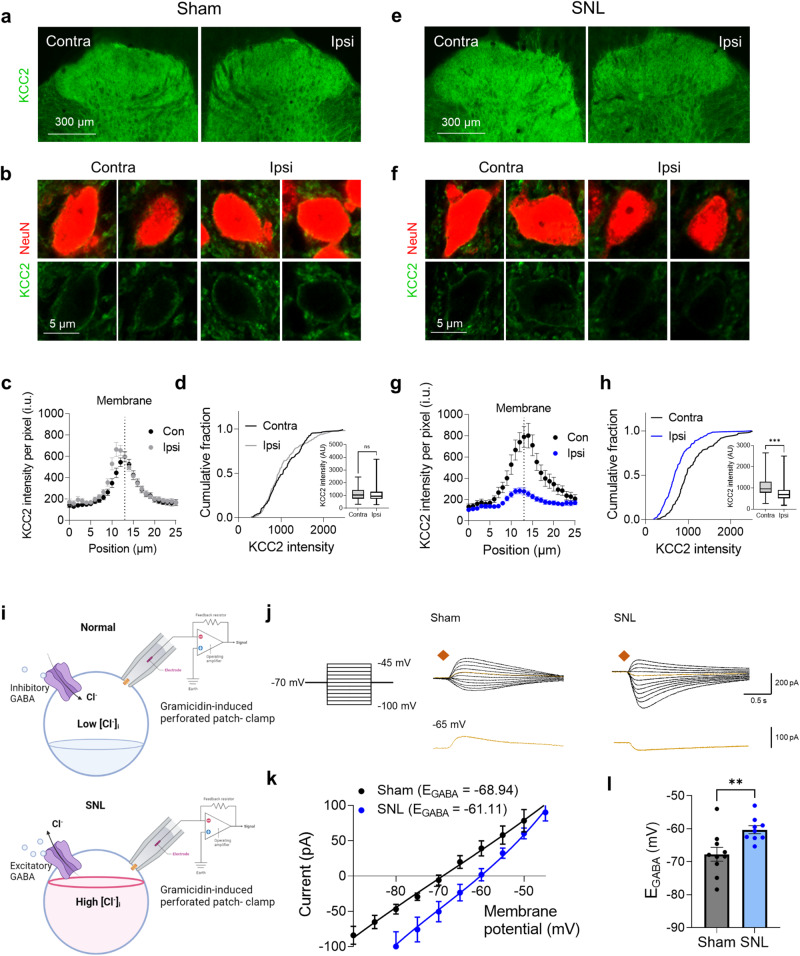
E_GABA_ is elevated as a result of reduced KCC2 expression in spinal dorsal horn neurons. **a** Immunohistochemistry of KCC2 in the ipsilateral and contralateral dorsal horns of the L5 segment of the spinal cord in sham-operated rats (*n* = 3 rats). **b** Magnified confocal images of neuronal KCC2 expression in the SDH neurons of sham-operated rats. **c** Membrane analysis of KCC2 intensity with respect to the distance to the membrane profile of the ipsilateral and contralateral spinal cords of sham-operated rats. **d** Cumulative fractions of neuronal KCC2 intensity compared with those of the ipsilateral and contralateral spinal cords of sham-operated rats. Inset, the mean intensity of KCC2 in neurons (Mann‒Whitney test, *p* = 0.1576). **e** Immunohistochemical analysis of KCC2 in the ipsilateral and contralateral dorsal horns of the L5 segment of the spinal cord in SNL-operated rats (*n* = 3 rats). **f** Magnified confocal images of neuronal KCC2 expression in the SDH neurons of SNL-operated rats. **g** Membrane analysis of KCC2 intensity with respect to the distance to the membrane profile of the ipsilateral and contralateral spinal cords of SNL-operated rats. **h** Cumulative fractions of neuronal KCC2 intensity compared with those of the ipsilateral and contralateral spinal cords of SNL-operated rats. Inset, the mean intensity of KCC2 in neurons (Mann‒Whitney test, *p* < 0.001). **i** Schematic diagram of the GPP technique and hypothetical internal chloride concentration in sham-operated (top) and SNL-operated rats (bottom). **j** Representative Cl^−^ currents induced by GABA (1 mM) puffing. The cell was voltage clamped at −70 mV, and voltage steps were applied in 5 mV increments from −100 to 45 mV (the square indicates the time of GABA puffing). **k** I‒V curve for the E_GABA_ in sham and SNL-operated rats. The E_GABA_ was -68.94 mV in the sham-operated rats and −61.11 mV in the SNL-operated rats (*n* = 4 rats for each group). **l** Bar graph of the E_GABA_ in sham and SNL-operated rats (two-tailed unpaired *t*-test, *p* = 0.0083). Sham (*n* = 10) and SNL (*n* = 9) groups. The error bars represent the means ± SEMs. *p < 0.05, **p < 0.01, ***p < 0.001, ns non-significant. For detailed statistical information, see also Supplementary Table 1.

Under most physiological conditions, GABA acts as an inhibitory transmitter through GABA_A_ receptor-mediated Cl^−^ influx. The direction of Cl^−^ movement across the membrane is determined by the reversal potential of Cl^−^, which is dependent on the balance between the intracellular and extracellular Cl^−^ concentrations. This balance is regulated by KCC2 and NKCC1 transporters^30,31^. Previous studies have shown that KCC2 expression is reduced in neuropathic pain models^5,8^ and is associated with microglial reactivity. However, NKCC1 expression remains unchanged^32,33^. To investigate this further, we performed immunohistochemistry to examine KCC2 expression in the neurons of the L5-level dorsal horn. We found that KCC2 was mainly localized along the plasma membrane of neurons (Fig. 4a, b). We then used line plot analysis to measure the KCC2 intensity on the plasma membrane and found that KCC2 expression levels were significantly lower in the ipsilateral dorsal horn than in the contralateral dorsal horn of the SNL-operated rats (Fig. 4e–h). However, sham-operated rats did not show any difference in KCC2 levels between the ipsilateral and contralateral dorsal horns (Fig. 4a–d). Additionally, we found a significant reduction in KCC2 intensity in the ipsilateral dorsal horn compared to the contralateral dorsal horn in the SNL model rats treated with a MAOB inhibitor, a pattern consistent with that in the SNL model rats not treated with the MAOB inhibitor (Supplementary Fig. 4). This finding indicates that pharmacological blockade of MAOB did not restore the reduced KCC2 levels in the SNL-operated rats. Taken together, these findings raise the possibility that reduced KCC2 expression could result in an increase in intracellular Cl^−^ levels, leading to a dramatic change in the signaling of GABA from inhibitory to excitatory.

To investigate this possibility, we conducted gramicidin-perforated patch clamp experiments on neurons located in the ipsilateral dorsal horn layer II-III of acutely prepared L5 spinal cord sections from both sham-operated and SNL-operated rats. Using this method, we applied voltage steps to the neurons while puffing GABA (1 mM) next to them, allowing us to record the GABA-induced inward or outward Cl^−^ current without disturbing the internal Cl^−^ levels and to estimate the reversal potential of GABA (E_GABA_) (Fig. 4i). In sham-operated rats, we found that the E_GABA_ was approximately −68.94 mV, whereas in SNL-operated rats, there was a significant depolarizing shift of approximately 7.8 mV in the E_GABA_ (Fig. 4j‒l). Specifically, in the sham-operated rats, GABA induced an outward Cl^−^ current at the resting membrane potential (approximately −65 mV), indicating that GABA caused hyperpolarization and could function as an inhibitory transmitter. Conversely, in the SNL-operated rats, GABA induced an inward Cl^−^ current, indicating that GABA caused depolarization and could function as an excitatory transmitter. Taken together, these results suggest that the KCC2-mediated depolarizing shift in E_GABA_ facilitates the excitatory action of GABA.

### Paradoxically, astrocytic GABA excites neuronal firing

**Fig. 5:**
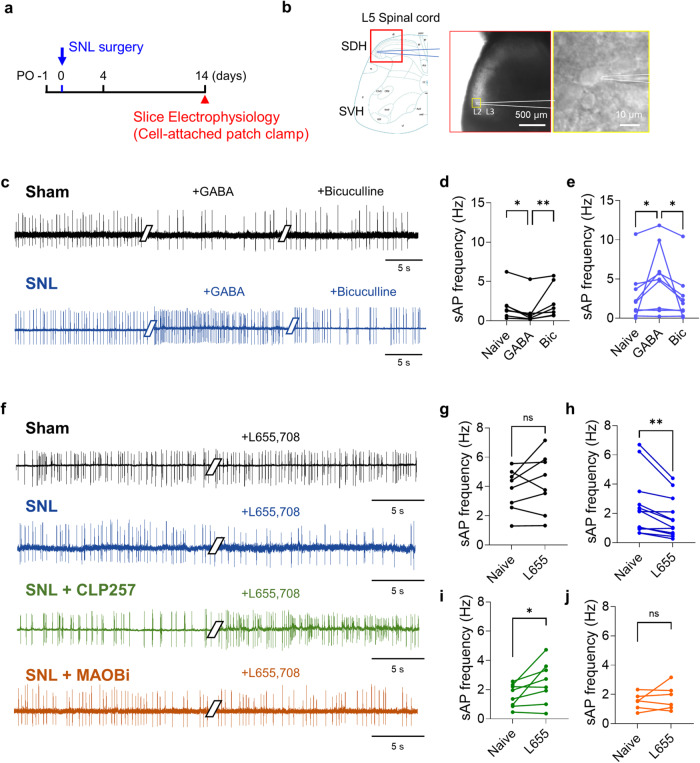
Astrocytic GABA paradoxically activates neuronal action potential firing in the SNL model. **a** Experimental timeline of spontaneous action potential (sAP) firing recorded from spinal dorsal horn neurons at the L5 segment. **b** Left, spinal dorsal horn (SDH) as the ROI. Right, representative image of a cell-attached patch clamp in SDH layer II. **c** Representative traces of sAP firing after bath application of GABA (10 µM) and bicuculine (50 µM) in sham and SNL-operated rats (*n* = 3 rats for each group). **d** Quantification of the sAP frequency before and after GABA and bicuculline treatments in sham-operated rats (Friedman test with Dunn’s multiple comparison test, *p* = 0.0003, *n* = 7). **e** Quantification of the sAP frequency before and after GABA and bicuculline treatments in SNL-operated rats (one-way ANOVA with Tukey’s multiple comparison test, *p* = 0.0217, *n* = 9). **f** Representative traces of sAP firing after bath application of L655,708 (10 µM) in the sham, SNL, and SNL groups preincubated with CLP257 (5 µM) or MAOBi (100 nM) (*n* = 3 or 4 rats for each group). **g** Quantification of sAP frequency before and after L655,708 treatment in sham-operated rats (two-tailed paired *t*-test, *p* = 0.2862, *n* = 8). **h** Quantification of sAP frequency before and after L655,708 treatments in SNL-operated rats (two-tailed paired *t*-test, *p* = 0.0050, *n* = 12). **i** Quantification of the sAP frequency before and after L655,708 treatment in SNL + CLP257 rats (two-tailed paired *t*-test, *p* = 0.0488, *n* = 8). **j** Quantification of sAP frequency before and after L655,708 treatments in SNL+MAOBi rats (two-tailed paired *t*-test, *p* = 0.4107, *n* = 6). The error bars represent the means ± SEMs. *p < 0.05, **p < 0.01, ***p < 0.001, ns non-significant. For detailed statistical information, see also Supplementary Table 1.

Next, we tested whether GABA augments neuronal firing in a neuropathic animal model. We performed cell-attached patch recordings to measure spontaneous action potential firing without disturbing the intracellular milieu (Fig. 5a, b). As expected, we found that in the sham-operated rats, the bath application of GABA (1 mM) decreased the firing rate of dorsal horn neurons, which was restored by the administration of the GABA_A_ receptor antagonist bicuculline (50 μM) (Fig. 5c, d). In contrast, in the SNL-operated rats, GABA significantly increased the firing rate of dorsal horn neurons, which was also reversed by bicuculline treatment (Fig. 5c, e). These findings indicate that the mode of GABA dramatically changes from inhibitory to excitatory in SNL-operated rats.

We demonstrated that increased astrocytic GABA synthesis tonically affects neighboring neurons in the ipsilateral dorsal horn of SNL-operated rats (Fig. 3). It has been well documented that astrocytic GABA, but not neuronal GABA, targets extrasynaptic GABA_A_ receptors, which mainly contain the a5 subunit. Thus, to determine whether this excessive astrocytic GABA tonically excites or inhibits neighboring neurons, we recorded spontaneous action potential firing with or without L655,708, an inverse agonist of the GABA_A_ receptor a5 subunit^34^, instead of bicuculline (Fig. 5f). In the sham-operated rats, L655,708 treatment did not alter the firing rate, unlike bicuculline treatment (Fig. 5f, g). This discrepancy is attributed to the low level of astrocytic GABA that binds to extrasynaptic GABA_A_ receptors under physiological conditions, while neuronal GABA mainly regulates neuronal firing. However, in the SNL-operated rats, L655,708 (10 μM) significantly suppressed the firing frequency, indicating that the excitatory action of extrasynaptic GABA mainly originates from astrocytes (Fig. 5f, h). Additionally, preincubation of spinal cord slices with CLP257 (5 μM), a KCC2 enhancer, restored the abnormal function of GABA from excitatory to inhibitory in SNL-operated rats (Fig. 5f, i) by decreasing the intracellular Cl^−^ concentration through the enhanced action of KCC2. Interestingly, dorsal horn neurons preincubated with MAOBi (100 nM) showed no change in firing rate upon L655,708 treatment (Fig. 5f, j) because the aberrant synthesis of astrocytic GABA was blocked, nullifying extrasynaptic GABA_A_ receptor-mediated tonic excitation of neighboring neurons. Overall, our results suggest that astrocytic GABA paradoxically activates dorsal horn neuronal action potential firings in a neuropathic pain model and may amplify or maintain pain signals. In addition, neuronal firing could be recovered by MAOBi treatment, indicating that astrocytic GABA release can be a proper target for treating neuropathic pain.

### SNL increases regional glucose metabolism in a MAOB-dependent manner

**Fig. 6:**
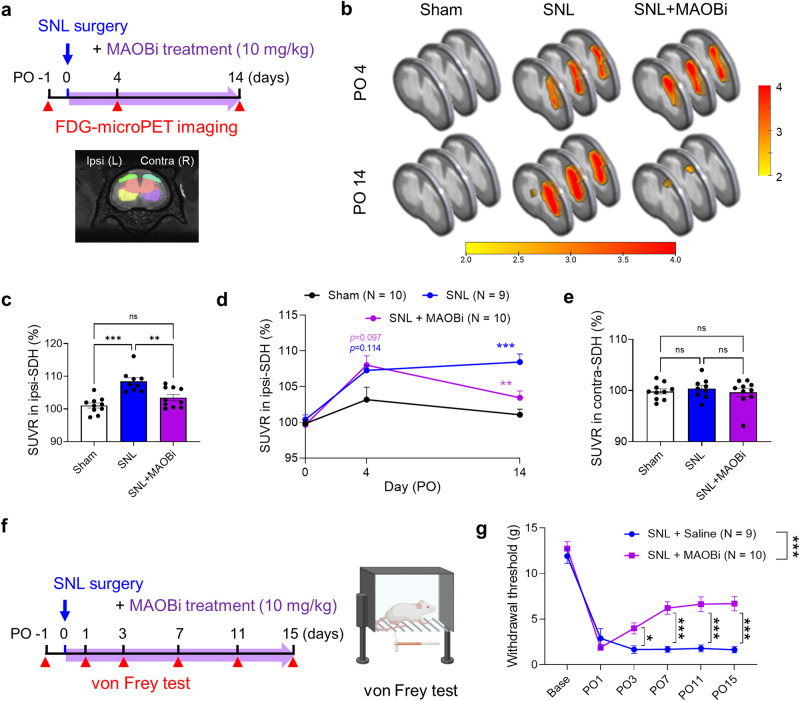
MAOB inhibition reversed the increase in glucose metabolism in the ipsilateral dorsal horn and neuropathic pain. **a** Schematic diagram and experimental timeline of ^18^F-FDG-microPET scans. **b** Representative ^18^F-FDG-microPET coronal images of sham, SNL, and SNL + MAOBi rats at PO4 and PO14. **c** Quantification of the SUVR of ^18^F-FDG uptake in the ipsilateral dorsal horn of the spinal cord (one-way ANOVA with Tukey’s multiple comparison test, F (2, 26) = 0.3380, *p* < 0.001). Sham (*N* = 10), SNL (*N* = 9), SNL + MAOBi (*N* = 9). **d** Changes in the SUVR in the ipsilateral dorsal horn on PO0, 4, and 14 in sham, SNL, and SNL + MAOBi rats (two-way ANOVA with Tukey’s multiple comparisons test, interaction, F (4, 52) = 3.889, *p* = 0.0077; time, F (1.586, 41.23) = 26.82, *p* < 0.0001; group, F (2, 26) = 13.16, *p* = 0.0001; *N* = 10, 9, and 10 rats for the sham, SNL, and SNL + MAOBi groups, respectively). **e** Quantification of the SUVR of ^18^F-FDG uptake in the contralateral dorsal horn of the spinal cord (one-way ANOVA with Tukey’s multiple comparison test, F (2, 26) = 0.2123, *p* = 0.7292). Sham (*N* = 10), SNL (*N* = 9), SNL+MAOBi (*N* = 9). **f** Experimental timeline and schematic diagram of consecutive von Frey tests with MAOBi treatment. **g** Mechanical withdrawal thresholds assessed by the von Frey test in SNL + saline (veh) and SNL + MAOBi rats on PO1, 3, 7, 11, and 15 (two-way ANOVA with Tukey’s multiple comparisons test, F (5, 85) = 8.396, *p* < 0.0001; time, F (3.680, 62.55) = 73.52, *p* < 0.0001; group, F (1, 17) = 31.12, *p* < 0.0001; *N* = 9, and 10 rats for the SNL + saline (veh) and SNL + MAOBi groups, respectively). The error bars represent the means ± SEMs. *p < 0.05, **p < 0.01, ***p < 0.001, ns non-significant. For detailed statistical information, see also Supplementary Table 1.

To deepen the understanding of the progression of diseases, including neuropathic pain, and patient prognosis, it is beneficial to monitor regional metabolic activity using noninvasive functional imaging strategies such as FDG-microPET^35,36^. It has been well documented that glucose metabolism is tightly correlated with neuronal excitability^15,27,37^. Therefore, we performed FDG-microPET scans to examine whether regional glucose metabolism is increased in the spinal dorsal horn of SNL-operated rats along with increased neuronal excitability (Fig. 6a). We found that the standardized uptake value ratio (SUVR) of 18F-FDG in the ipsilateral dorsal horn of the L5 spinal cord significantly increased from PO4 to PO14, indicating increased regional glucose metabolism (Fig. 6b‒d, Supplementary Fig. 5a, b). However, no significant alterations were observed in the sham-operated rats (Fig. 6b‒d, Supplementary Fig. 5a, b). Regional glucose metabolism was also significantly increased in the ipsilateral ventral horn, albeit to a lesser degree than that observed in the dorsal horn (Supplementary Fig. 5c‒f). On the other hand, an analogous increase in regional glucose metabolism was not discernible on the contralateral side of the spinal cord. (Fig. 6e). These results indicate a concurrence of glucose hypermetabolism and reactive gliosis within the ipsilateral dorsal and ventral horns. Our findings contrast with those of previous studies, which demonstrated that reactive astrogliosis can lead to a significant decrease in regional glucose metabolism in the brains of patients with Alzheimer’s disease and stroke^15,27^. This discrepancy is likely due to the opposing modes of action of astrocytic GABA in Alzheimer’s disease and stroke (inhibitory) and neuropathic pain (excitatory).

Next, we investigated whether the astrocytic GABA-mediated tonic excitation of neighboring neurons is indeed responsible for glucose hypermetabolism in the spinal dorsal horn of SNL-operated rats. We found that intraspinal treatment with MAOBi via an osmotic pump for 2 weeks significantly decreased glucose metabolism in the ipsilateral dorsal horn. This finding is consistent with decreased glial reactivity and the associated attenuation of neuronal excitability (Fig. 6d, Supplementary Fig. 5a, b). Similar features were observed in the ipsilateral ventral horn (Supplementary Fig. 5c, d). Conversely, MAOBi did not induce any significant change in the SUVR on the contralateral side (Fig. 6e). These results indicate that MAOB-dependent astrocytic GABA is critical for the increase in regional glucose metabolism in the spinal cord in neuropathic pain model rats.

### Blockade of astrocytic GABA synthesis reverses mechanical allodynia

Finally, we tested whether mechanical allodynia can be alleviated by MAOBi-mediated blockade of astrocytic GABA synthesis (Fig. 6f). Through the von Frey test, we found that MAOBi treatment significantly restored the reduced withdrawal threshold in the neuropathic pain model rats, consistent with its ability to alleviate reactive astrogliosis and the associated tonic excitation (Fig. 6g). These findings suggest that MAOBi may have a potential therapeutic effect on mechanical allodynia. Notably, MAOBi-mediated recovery of mechanical allodynia is associated with the attenuation of glucose hypermetabolism in the ipsilateral spinal cord. Hence, FDG-PET imaging shows great potential for visualizing progressive pathology and assessing the effectiveness of treatments in clinical settings, thereby aiding in determining the prognosis of patients with neuropathic pain. Overall, our study provides evidence that SNL induces aberrant GABA synthesis in reactive astrocytes, which leads to the tonic excitation of neighboring dorsal horn neurons. This is mediated by a significant decrease in KCC2 expression causing impairment of Cl^−^ homeostasis and dysfunctional GABAergic signaling. Blockade of astrocytic GABA synthesis attenuates mechanical allodynia, suggesting that this strategy can be an appropriate target for controlling neuropathic pain.

## Discussion

Despite the increasing recognition of the importance of glial cells and their possible contribution to the initiation and persistence of neuropathic pain, there remains a need for comprehensive investigations into the underlying astrocyte-mediated molecular and cellular mechanisms, as well as their potential therapeutic implications in neuropathic pain^23^. This study demonstrated that SNL downregulates the expression of the KCC2 transporter, resulting in dysfunctional GABA signaling and consequential functional shifts in which GABA transitions from an inhibitory role to an excitatory role. We also found that reactive astrocytes synthesize an excessive amount of GABA in the spinal dorsal horn following SNL. Consequently, this astrocytic GABA has been shown to paradoxically excite neighboring neuronal firings via SNL-induced KCC2 downregulation, thereby critically contributing to the development and persistence of mechanical allodynia. FDG-microPET imaging revealed hypermetabolism in both the dorsal and ventral horns, suggesting increased neuronal excitability. This neuronal hyperexcitability can be attributed to two key factors: excessive GABA release from reactive astrocytes and decreased KCC2 transporter expression, both of which are associated with neuroinflammation. Notably, this study demonstrated that the pharmacological blockade of astrocytic GABA synthesis by MAOBi can reverse mechanical allodynia and glucose hypermetabolism in the spinal cord. These findings provide novel insights into the pathogenesis of neuropathic pain and suggest that glial modulation through the blockade of astrocytic GABA synthesis could be a potential therapeutic strategy for controlling neuropathic pain.

Reactive astrogliosis has been increasingly recognized as a significant pathological factor and a promising therapeutic target for various neurological disorders involving neuroinflammation^15,16,29,38–40^. Our previous studies showed that reactive astrocytes excessively synthesize and release GABA, leading to the aberrant tonic inhibition of neighboring neurons in various brain regions^15,16,39^. In this study, we demonstrated that the depolarized reversal potential of GABA due to reduced KCC2 expression in the neurons of the spinal dorsal horn is responsible for the dramatic transition of the role of astrocytic GABA from inhibitory to excitatory^33^. Our findings indicate that astrocytic GABA can aberrantly increase neighboring neuronal activity in this specific situation, as shown in other conditions such as epilepsy where GABA-containing reactive astrocytes and increased neuronal excitability coexist. In this study, we intrathecally administered MAOBi to block the aberrant synthesis of astrocytic GABA and nullify the extrasynaptic GABA_A_ receptor-mediated tonic excitation of neighboring neurons. This intervention led to a reduction in mechanical allodynia and a corresponding decrease in tonic GABA-mediated excitation of spinal dorsal horn neurons. Based on these findings, we propose an unprecedented concept of tonic excitation mediated by astrocytic GABA as a core pathology and a potential therapeutic target for neuropathic pain and perhaps even for epilepsy.

Neuropathic pain is often intractable despite the administration of potent analgesics, such as opioids, anticonvulsants, and antidepressants. To circumvent this impediment, recent studies have focused the use of KCC2 enhancers, such as CLP257, to upregulate the expression of the KCC2 transporter, which shows promise in mitigating mechanical hypersensitivity and sensory neuronal excitability^41^. However, KCC2 enhancers may have off-target effects on other chloride channels, which could lead to unintended consequences and side effects^41,42^. Furthermore, long-term systemic manipulation of KCC2 expression leads to the disruption of chloride homeostasis and affects neuronal functions and plasticity in brain regions. Moreover, several lines of evidence supporting the role of astrocytes in neuropathic pain^11,13,14^ have inspired the development of drug candidates targeting astrocytes, although limited known targets within the astrocyte. To date, two major strategies targeting astrocytes have been employed: suppressing the release of astrocyte-derived inflammatory factors by targeting MAPK signaling, purinergic receptors, or hemichannels and modulating downstream signaling mediated by chemokines and cytokines^23,43–45^. However, these approaches have consistently encountered challenges, including low therapeutic efficacy and undesirable side effects^46^. Here, our study proposes an alternative approach by specifically targeting MAOB-mediated astrocytic GABA synthesis, which is critical for aberrantly increasing the firing of spinal dorsal horn neurons and mechanical allodynia. Given the extensive clinical use and proven safety of several kinds of MAOBi, we suggest that MAOBi could be a safe and efficacious therapeutic option for neuropathic pain management.

Predicting patient prognosis and monitoring the therapeutic efficacy of drugs for neuropathic pain can substantially aid its treatment in clinical practice. For this purpose, we aimed to obtain regional metabolic images of the spinal cord corresponding to the level of the damaged spinal nerve root, where the primary pathological event occurs. Previous studies have reported increased glucose uptake in various brain regions, including the somatosensory cortex, thalamus, anterior cingulate cortex, and hippocampus, in neuropathic pain model rats and human patients^47^. However, these images were obtained mostly from the brain and are therefore insufficient to explain pathological events in the spinal cord. Moreover, other studies have shown that metabolic activity initially increases and then decreases following spinal cord contusion^48,49^. However, in the SNL model, metabolic activity persistently increased. Thus, this imaging approach was useful for revealing the unique metabolic changes in the spinal cord in patients with neuropathic pain induced by SNL.

Previous studies have presented conflicting evidence regarding the impact of neuroinflammation and reactive gliosis on regional glucose metabolism. Some studies have demonstrated that surgery-induced neuroinflammation acutely increases glucose metabolism, possibly due to reactive microglia^48–50^. On the other hand, other studies, including ours, have reported that neuroinflammation-related reactive astrocytes release an aberrant amount of GABA, resulting in reduced neuronal activity and decreased glucose demand, ultimately leading to a significant decline in regional metabolic activity in conditions such as stroke, Alzheimer’s disease, and adenovirus-induced neuroinflammation^15,27^. In the present study, we observed increased glucose metabolism in the spinal dorsal horn, which is less likely due to surgery-induced acute reactions because glucose metabolism persisted for at least two weeks after SNL. Instead, based on previous reports demonstrating that glucose metabolism is tightly associated with neuronal activity^15,27,37^, this is likely due to increased neuronal activity through dysfunctional GABA induced by KCC2 downregulation in our SNL model. Notably, these findings contrast with our previous findings, which revealed that astrocytic GABA inhibited neuronal activity in stroke and Alzheimer’s disease, leading to glucose hypometabolism^15,27^. Thus, these findings suggest that metabolic imaging reflects the excitability of the spinal cord induced by excessive astrocytic GABA and KCC2 downregulation.

Most importantly, we found that mechanical allodynia significantly correlated with increased glucose metabolism in the ipsilateral spinal cord in our SNL model. Furthermore, we observed a significant decrease in regional glucose metabolism in the spinal cord, which correlated with a decrease in mechanical allodynia following MAOBi administration and reversed the tonic excitation of GABA. The ability to longitudinally monitor changes in glucose metabolism in the spinal cord provides a new approach for evaluating the therapeutic efficacy of potential treatments for neuropathic pain in vivo.

In conclusion, chronic neuropathic pain can arise due to tonic excitation by astrocytic GABA, which is mediated by KCC2 dysfunction, in the spinal dorsal horn. Astrogliosis, which occurs following nerve injury, leads to the excessive production of astrocytic GABA, further driving pathological neuronal hyperexcitability. This study highlights the importance of astrocytic GABA and its contribution to mechanical allodynia. Notably, the blockade of astrocytic GABA synthesis using MAOBi can reduce abnormally increased neuronal hyperexcitability and improve dysfunctional GABA signaling, ultimately reducing mechanical allodynia. Additionally, our application of FDG-microPET imaging provided insight into regional glucose metabolism in the spinal cord during neuropathic pain conditions. The hypermetabolism detected in these images was closely correlated with the presence of mechanical allodynia and excessive excitatory GABA released by reactive astrocytes. Moreover, the reduction in hypermetabolism is coupled with improved mechanical allodynia and dysfunctional GABA signaling. Thus, FDG-microPET is a useful tool for longitudinally monitoring spinal cord excitability and estimating therapeutic efficacy in vivo. Taken together, our findings suggest that modulating the synthesis of astrocytic GABA could be a promising therapeutic approach for managing neuropathic pain.

### Supplementary information

Supplementary information

Dataset for all figures

## Data Availability

The data supporting the findings from this study are available within the manuscript and its Supplementary Information. Source data for every graph are provided in this paper.
