# Author Correction: Tonic excitation by astrocytic GABA causes neuropathic pain by augmenting neuronal activity and glucose metabolism

**DOI:** 10.1038/s12276-024-01306-y

**Published:** 2024-08-30

**Authors:** Yeon Ha Ju, Jongwook Cho, Ji-Young Park, Hyunjin Kim, Eun-Bin Hong, Ki Duk Park, C. Justin Lee, Euiheon Chung, Hyoung-Ihl Kim, Min-Ho Nam

**Affiliations:** 1 Brain Science Institute, Korea Institute of Science and Technology (KIST), Seoul, 02792 Republic of Korea; 2 Department of Biomedical Science and Engineering, Gwangju Institute of Science and Technology (GIST), Gwangju, 61005 Republic of Korea; 3 Center for Cognition and Sociality, Institute for Basic Science, Daejeon, 34126 Republic of Korea; 4 Department of Neurosurgery, Presbyterian Medical Center, Jeonju, 54987 Republic of Korea; 5 Department of KHU-KIST Convergence Science and Technology, Kyung Hee University, Seoul, 02447 Republic of Korea

**Keywords:** Astrocyte, Neurotransmitters, Neuropathic pain

Correction to: *Experimental & Molecular Medicine* 10.1038/s12276-024-01232-z, published online 17 May 2024

After online publication of this article, the authors noticed an error in the author list.

“Ki Duk Park” should have been inserted as a co-author to the author list and “Center for Brain Function” should be removed from the Affiliation 1.

The authors apologize for any inconvenience caused.

The original article has been corrected.

