# Supplementary material for: Tonic excitation by astrocytic GABA causes neuropathic pain by augmenting neuronal activity and glucose metabolism
Source: Exp Mol Med. 2024 May 17;56(5):1193–205. doi: 10.1038/s12276-024-01232-z (PMC11148027; doi:10.1038/s12276-024-01232-z)
Supplement: Supplementary file 1 — Supplementary information [file 12276_2024_1232_MOESM1_ESM.pdf]

Supplementary Information

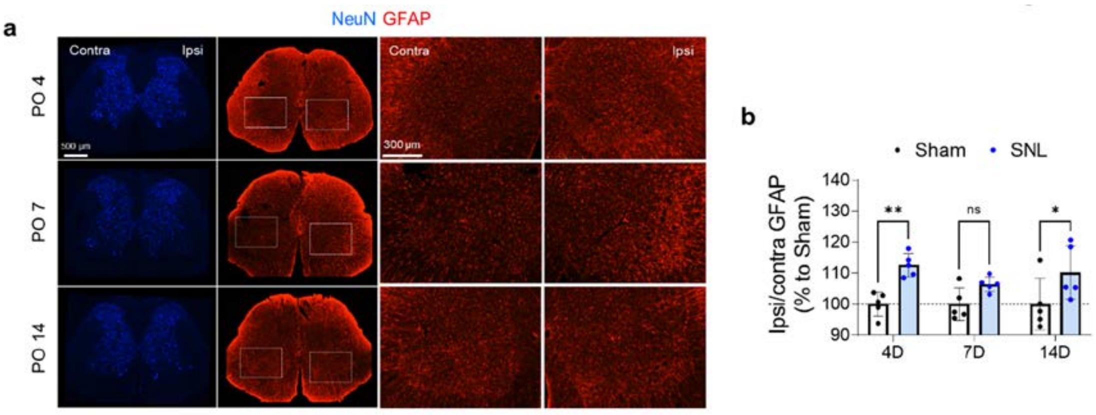

Supplementary Fig. 1. Reactive astrocytes in the spinal ventral horn in SNL model.

a, Immunohistochemistry (NeuN and GFAP) of spinal cord ventral horn of L5 segments for SNL-operated rats on PO 4, 7, and 14 (Scale bar = 500 μm). Right, magnified view of ventral horn region (Scale bar = 300 μm). **b,** Bar graphs of quantification of GFAP intensity of ipsilateral ventral horn at PO 4, 7, and 14 (Two-way ANOVA with Sidak's multiple comparisons test, Time F (2, 24)   0.7006, $p = 0.5062$; Group F (1, 24) = 20.61, $p = 0.0001$), N = 5 for each group. Error bars represent means ± SEM. *$p < 0.05$, **$p < 0.01$, ***$p < 0.001$, ns, non-significant.

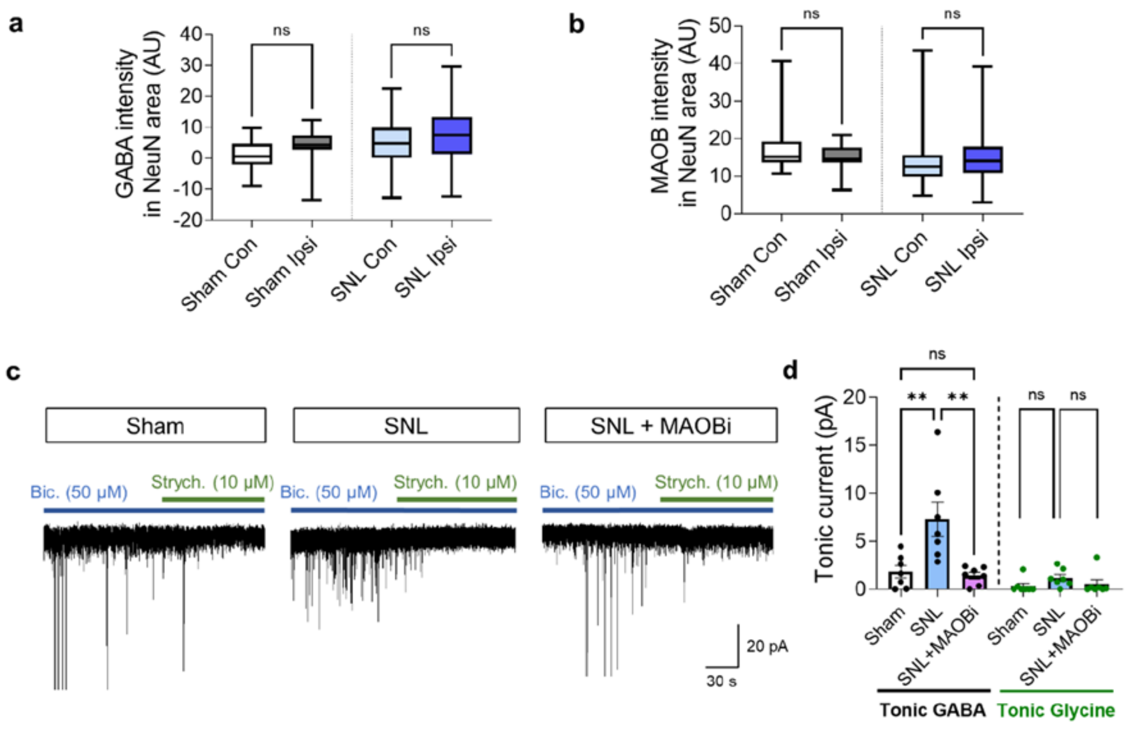

Supplementary Fig. 2. No change in neuronal expressions of GABA and MAOB, and tonic glycine current.

a, Quantification of GABA intensity in NeuN-positive neurons in the dorsal horn of Sham and SNL groups at PO 14 (Brown-Forsythe one-way ANOVA with Dunnett T3 multiple comparison test, F (3.000, 231.5) = 8.784, p < 0.0001). **b,** Quantification of MAOB intensity in NeuN-positive neurons in the dorsal horn of Sham and SNL groups at PO 14 (Brown-Forsythe one-way ANOVA with Dunnett T3 multiple comparison test, F (3.000, 130.1) = 3.116, p = 0.0285). **c,** Representative traces of tonic GABA and glycine currents revealed by bath application of bicuculline (50 μM) and strychnine (10 μM), respectively. **d,** Quantification of tonic GABA and tonic glycine currents (Tonic GABA, one-way ANOVA with Tukey's multiple comparison test, F (2, 18) = 8.886, p = 0.0021; tonic glycine, one-way ANOVA with Tukey's multiple comparison test, F (2, 18) = 1.411, p = 0.2695), n = 7 for each group. Error bars represent means ± SEM. *p < 0.05, **p < 0.01, ***p < 0.001, ns, non-significant.

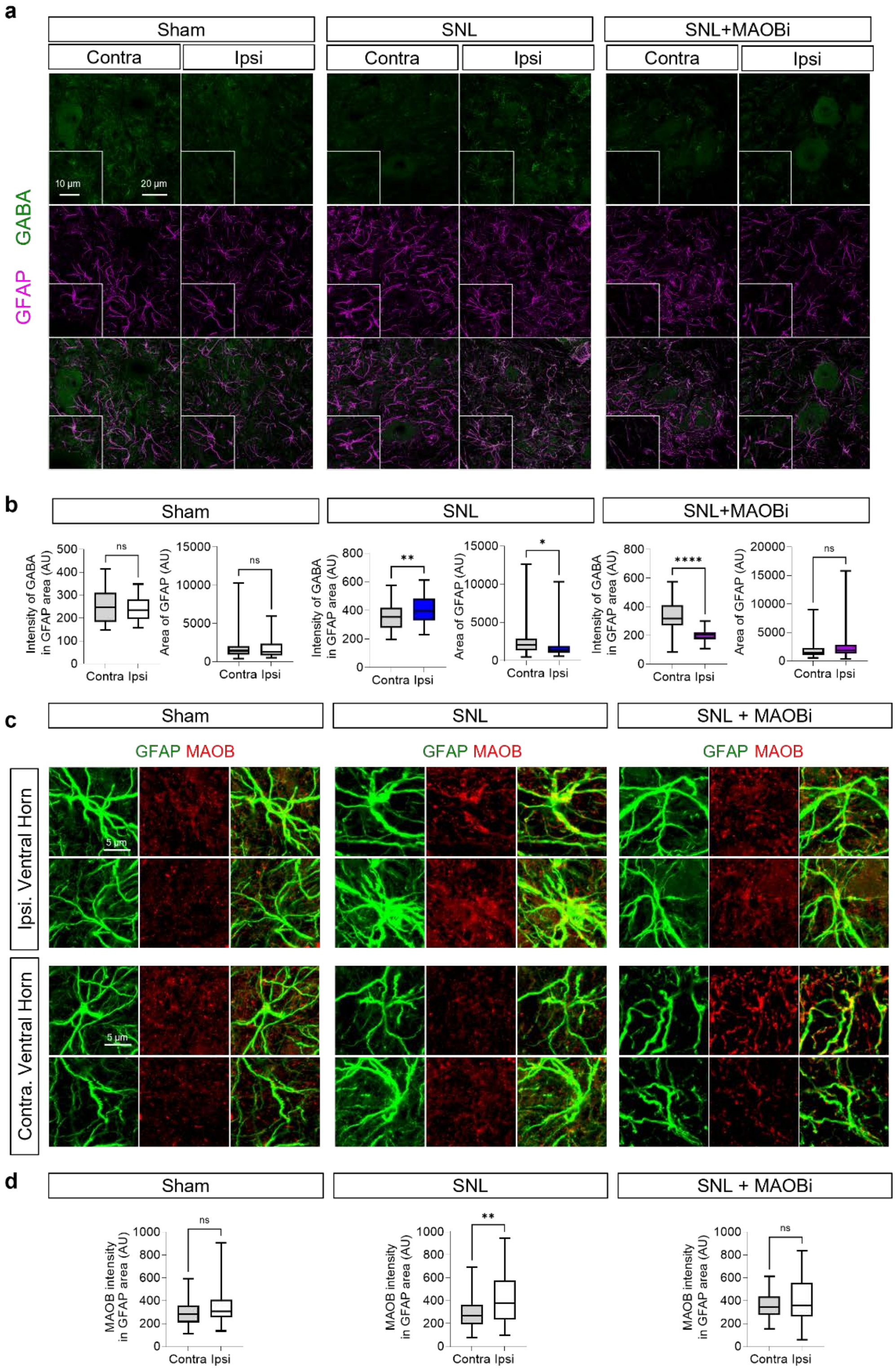

Supplementary Fig. 3. Astrocytic GABA and MAOB are upregulated in the ipsilateral ventral horn in SNL model.

a, Representative fluorescent images of GFAP and GABA-stained spinal ventral horn tissues. **b,** Quantification of GABA intensity in GFAP-positive astrocytes and area of GFAP-positive pixels in the spinal dorsal horn of Sham, SNL, and SNL+MAOBi groups at PO 14 (Sham: GABA intensity, two-tailed unpaired t-test with Welch's correction, p = 0.3250; Area of GFAP, two-tailed unpaired t-test with Welch's correction, p = 0.6044. SNL: GABA intensity, two-tailed unpaired t-test, p = 0.0043; Area of GFAP, two-tailed unpaired t-test, p = 0.0479; SNL+MAOBi: GABA intensity, t wo-tailed unpaired t-test with Welch's correction, p = 0.0001; Area of GFAP, two-tailed unpaired t-test with Welch's correction, p = 0.1132). **c,** Representative fluorescent images of GFAP and MAOB-stained astrocytes in the spinal ventral horn of each group. **d,** Quantification of MAOB intensity in GFAP-positive astrocytes in the ventral horn of Sham, SNL, and SNL+MAOBi groups at PO 14 (Sham, two-tailed unpaired t-test, p = 0.2788; SNL, wo-tailed unpaired t-test with Welch's correction, p = 0.0028; SNL+MAOBi, t wo-tailed unpaired t-test with Welch's correction, p = 0.1279). Error bars represent means ± SEM. *p < 0.05, **p < 0.01, ***p < 0.001, ns, non-significant.

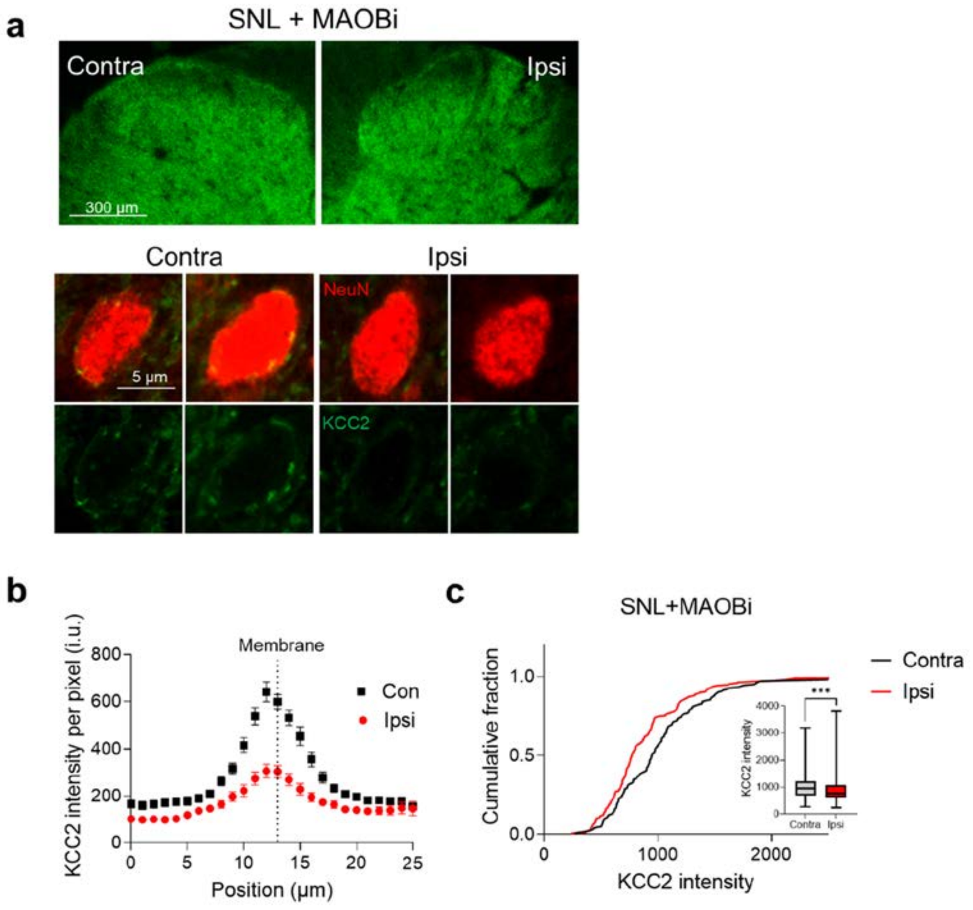

Supplementary Fig. 4. MAOB inhibition does not affect KCC2 expression in the dorsal horn. **a,** Representative fluorescence images of KCC2 in ipsilateral and contralateral of the spinal dorsal horn of SNL+MAOBi groups (Scale bar = 5 μm). **b,** Membrane analysis of KCC2 intensities with the distance to the membrane profile of ipsilateral and contralateral spinal cord of SNL+MAOBi rats (n = 35 and 42 for contralateral and ipsilateral side, respectively). **c,** Cumulative fractions of neuronal KCC2 intensity compared with ipsilateral and contralateral side (n = 35 and 42 for contralateral and ipsilateral side, respectively). Inset, the mean intensity of KCC2 in neurons (Unpaired t-test, p = 0.0003). Error bars represent means ± SEM. *p < 0.05, **p < 0.01, ***p < 0.001, ns, non-significant.

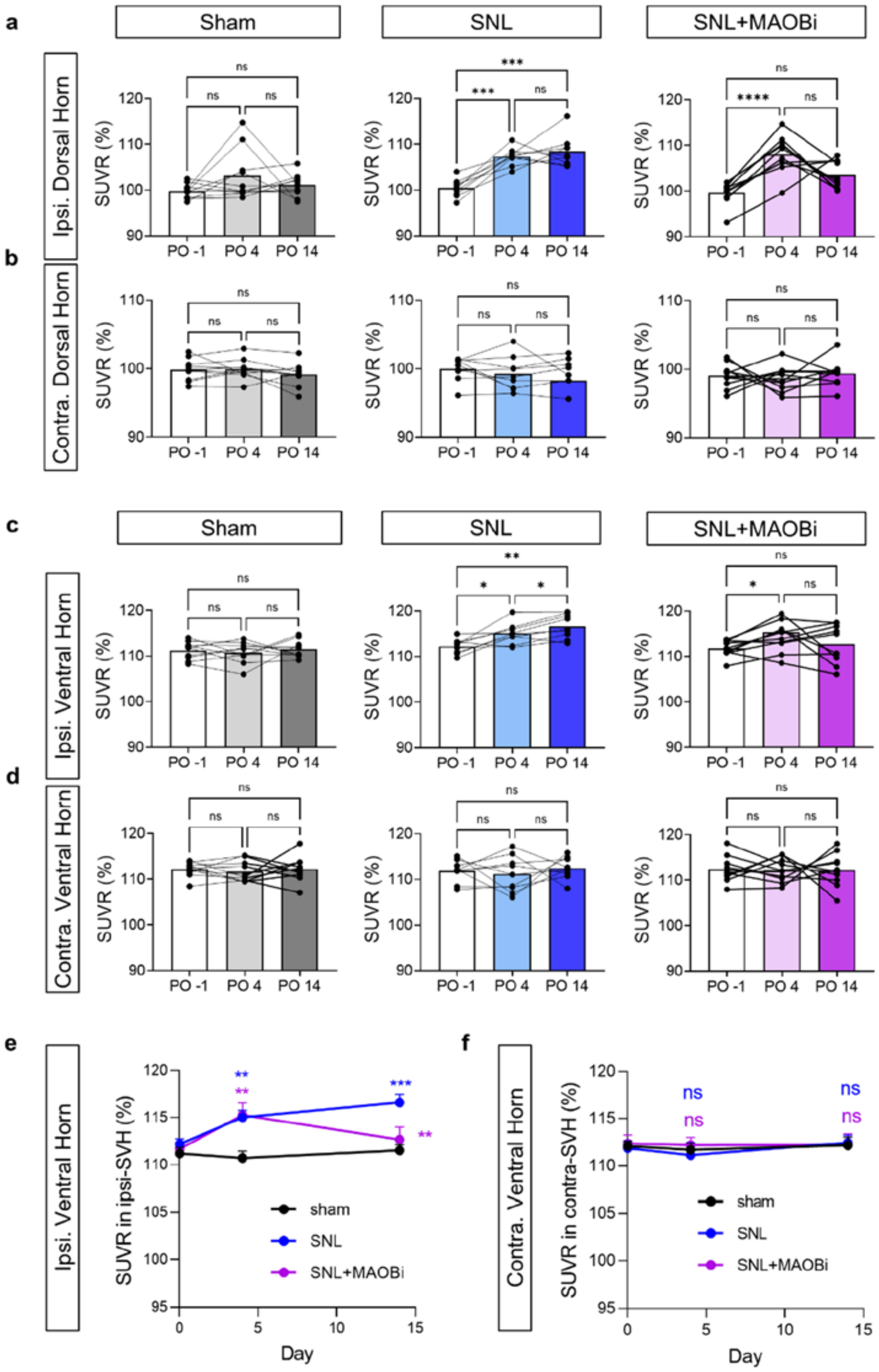

Supplementary Fig. 5. Glucose metabolism is up-regulated in the ipsilateral dorsal and ventral horn.

a, Normalized SUV of ^{18}F-FDG uptake in the ipsilateral dorsal horn of Sham, SNL and SNL+MAOBi rats on PO-1, 4, and 14. (Sham, N = 10, repeated measure one-way ANOVA with Tukey, $F_{(2, 18)} = 1.931$, $p = 0.1738$; SNL, N = 9, repeated measure one-way ANOVA adjusted

by Geisser-Greenhouse correction with Tukey $F(1.594, 12.75) = 27.41$, $p < 0.001$; SNL+MAOBi, N = 10, repeated measure one-way ANOVA adjusted by Geisser-Greenhouse correction with Tukey $F(1.225, 11.29) = 15.61$, $p = 0.0014$). **b,** Normalized SUV of ^{18}F-FDG uptake in the contralateral dorsal horn of Sham, SNL and SNL+MAOBi rats on PO-1, 4, and 14. (Sham, repeated measure one-way ANOVA with Tukey, $F(2, 18) = 1.398$, $p = 0.2726$; SNL, repeated measure one-way ANOVA with Tukey, $F(2, 24) = 0.7742$, $p = 0.4722$; SNL+MAOBi, repeated measure one-way ANOVA with Tukey, $F(2, 18) = 0.3045$, $p = 0.7412$). **c,** Normalized SUV of ^{18}F-FDG uptake in the ipsilateral ventral horn of Sham, SNL and SNL+MAOBi rats on PO-1, 4, and 14. (Sham, repeated measure one-way ANOVA with Tukey, $F(2, 18) = 0.4625$, $p = 0.6370$; SNL, repeated measure one-way ANOVA adjusted by Geisser-Greenhouse correction with Tukey $F(1.613, 12.90) = 17.70$, $p < 0.001$; SNL+MAOBi, repeated measure one-way ANOVA adjusted by Geisser-Greenhouse correction with Tukey, $F(1.872, 16.85) = 4.119$, $p = 0.0371$). **d,** Normalized SUV of ^{18}F-FDG uptake in the contralateral ventral horn of Sham, SNL and SNL+MAOBi rats on PO-1, 4, and 14 (Sham, repeated measure one-way ANOVA with Tukey, $F(2, 18) = 0.1768$, $p = 0.8394$. SNL, repeated measure one-way ANOVA with Tukey, $F(2, 16) = 0.3749$, $p = 0.6932$; SNL+MAOBi, repeated measure one-way ANOVA with Tukey, $F(2, 18) = 0.006218$, $p = 0.9983$). **e,** Time course of normalized SUV of ^{18}F-FDG uptake in the ipsilateral ventral horn (repeated measure one-way ANOVA with Tukey. Interaction, $F(4, 52) = 4.761$, $P=0.0024$. Time, $F(2, 52) = 7.446$, $p = 0.0014$. Group, $F(2, 26) = 7.190$, $p = 0.0033$. Subject, $F(26, 52) = 2.472$, $p = 0.0027$). **f,** Time course of normalized SUV of ^{18}F-FDG uptake in the contralateral ventral horn. (Repeated measure one-way ANOVA with Tukey. Interaction, $F(4, 52) = 0.1165$, $p = 0.9761$. Time, $F(1.604, 41.70) = 0.3292$, $p = 0.6736$. Group, $F(2, 26) = 0.2308$, $p = 0.7955$. Subject, $F(26, 52) = 0.7173$, $p = 0.8197$). Error bars represent means ± SEM. *p < 0.05, **p < 0.01, ***p < 0.001, ns, non-significant.

Supplementary Table 1. Detailed information of statistical analyses

Figure	Statistics
1b	Sham: Base (12.81 ± 0.6610), PO1 (7.507 ± 1.144), PO3 (8.325 ± 0.7396), PO7 (10.67 ± 1.173), PO11 (9.546 ± 0.8684), PO15 (9.810 ± 1.238)<br>SNL: Base (13.01 ± 0.8032), PO1 (2.898 ± 0.6446), PO3 (1.886 ± 0.2332), PO7 (2.087 ± 0.2272), PO11 (1.931 ± 0.3243), PO15 (1.926 ± 0.2384)<br>Two-way ANOVA with Bonferroni's multiple comparisons test Interaction<br>Interaction, $F (5, 85) = 11.71$, $p < 0.0001$; Time, $F (3.414, 58.04) = 39.49$, $p < 0.0001$; Group, F (1.17) = 61.60, $p < 0.0001$
1d	4D, Sham (100.000 ± 2.539), 4D, SNL (111.878 ± 1.665), 7D, Sham (100.000 ± 2.688), 7D, SNL (106.741 ± 1.072), 14D, Sham (100.000 ± 1.722), 14D, SNL (115.673 ± 4.057)<br>Two-way ANOVA with Sidak's multiple comparisons test<br>Interaction, Time $F (2, 24) = 1.628$, $p = 0.2172$; Time, $F (2, 24) = 1.628$, $p = 0.2172$; Group, $F (1, 24) = 31.76$, $p < 0.0001$
2b	GABA intensity: Contra (235.8 ± 15.43), Ipsi (252.9 ± 7.072), Unpaired t-test, $p = 0.2782$<br>GFAP area: Contra (905.9 ± 71.63), Ipsi (1082 ± 65.25), Unpaired t-test, $p = 0.0853$
2c	GABA intensity: Contra (355.6 ± 13.88), Ipsi (419.2 ± 8.849), Unpaired t-test with Welch's correction, $p < 0.001$<br>GFAP area: Contra (1382 ± 93.29), Ipsi (2326 ± 150.1), Unpaired t-test with Welch's correction, $p < 0.001$
2d	GABA intensity: Contra (200.6 ± 8.470), Ipsi (207.3 ± 6.497), Unpaired t-test with Welch's correction, $p = 0.5318$<br>GFAP area: Contra (2104 ± 30.47), Ipsi (2178 ± 39.59), Unpaired t-test with Welch's correction, $p = 0.1428$
2f	Sham: Con (446.6 ± 21.14), Ipsi (447.7 ± 17.46)<br>Two-tailed unpaired t-test, $p = 0.9669$
2g	SNL: Con (476.0 ± 18.85), Ipsi (605.2 ± 21.39)<br>Two-tailed unpaired t-test with Welch's correction, $p < 0.0001$
2h	SNL+MAOBi: Con (556.6 ± 20.98), Ipsi (539.7 ± 20.88)<br>Two-tailed unpaired t-test, $p = 0.5710$
3d	Con (0.5299 ± 0.1918), SNL (5.116 ± 0.8361), SNL+MAOBi (1.594 ± 0.5801)<br>Brown-Forsythe one-way ANOVA with Dunnett T3 multiple comparison test, $F (2.000, 23.36) = 19.24$, $p < 0.001$
3e, inset	Con (3.473 ± 0.3772), SNL (3.571 ± 0.5884), SNL+MAOBi (4.295 ± 0.415)<br>One-way ANOVA with Tukey's multiple comparison test, $F (2, 33) = 5.253$, $p = 0.6388$
3f, inset	Con (-18.65 ± 2.548), SNL (-28.29 ± 4.309), SNL+MAOBi (-23.22 ± 3.463)<br>One-way ANOVA with Tukey's multiple comparison test, $F (2, 33) = 1.698$, $p = 0.1987$
4d, inset	Con (1088 ± 34.83), Ipsi (1077 ± 37.21)<br>Unpaired t-test with Mann-Whitney test, $p = 0.3814$
4h, inset	Con (1080 ± 39.90), Ipsi (750.3 ± 24.77)<br>Unpaired t-test with Mann-Whitney test, $p < 0.001$
4l	Sham (-67.76 ± 2.095), SNL (-60.31 ± 1.219)<br>Unpaired t-test, $p = 0.0083$
5d	Naive (3.718 ± 1.338), GABA (2.359 ± 0.9620), Bic (3.441 ± 0.9129)<br>One-way ANOVA with Friedman test, $p = 0.0003$
5e	Naive (2.824 ± 1.103), GABA (5.065 ± 1.319), Bic (2.667 ± 1.062)<br>One-way ANOVA with Tukey's test, $p = 0.0217$
5g	Naive (3.787 ± 0.5047), L655,708 (4.265 ± 0.7027)<br>Paired t-test, $p = 0.2862$
5h	Naive (2.508 ± 0.5917), L655,708 (1.170 ± 0.4011)<br>Paired t-test, $p = 0.0050$
5i	Naive (1.591 ± 0.2754), L655,708 (2.483 ± 0.5031)<br>Paired t-test, $p = 0.0488$

5j	Naive (1.522 ± 0.2294), L655,708 (1.783 ± 0.3528)<br>Paired t-test, p = 0.4107
6c	Sham (101.1 ± 0.7776), SNL (108.4 ± 1.128), SNL+MAOBi (103.4 ± 0.9506)<br>One-way ANOVA with Tukey's multiple comparison test, F (2, 26) = 0.3380, p < 0.001
6d	Sham (101.1 ± 0.7776), SNL (108.4 ± 1.128), SNL+MAOBi (103.4 ± 0.9506)<br>Two-way ANOVA with Tukey's multiple comparisons test<br>Interaction F (4, 52) = 3.889, p = 0.0077; Time F (1.586, 41.23) = 26.82, p < 0.0001; Group F (2, 26) = 13.16, p = 0.0001
6e	Sham (99.82 ± 0.5004), SNL (100.4 ± 0.6510), SNL+MAOBi (99.65 ± 0.8164)<br>One-way ANOVA with Tukey's multiple comparison test, F (2, 26) = 0.2123, p = 0.7292
6g	SNL+saline: Base (11.92 ± 0.7906), PO1 (2.893 ± 1.058), PO3 (1.663 ± 0.4151), PO7 (1.689 ± 0.3140), PO11 (1.798 ± 0.3750), PO15 (1.644 ± 0.3321)<br>SNL+MAOBi: Base (12.74 ± 0.7676), PO1 (1.891 ± 0.3222), PO3 (3.989 ± 0.6873), PO7 (6.219 ± 0.6873), PO11 (6.645 ± 0.8028), PO15 (6.705 ± 0.7920)<br>Two-way ANOVA with Geisser-Greenhouse correction and Sidak's multiple comparisons test<br>Interaction F (5, 85) = 8.396, p < 0.0001; Time F (3.680, 62.55) = 73.52, p < 0.0001; Group F (1, 17) = 31.12, p < 0.0001
S1b	4D, Sham (100.000 ± 1.750), 4D, SNL (112.552 ± 1.675), 7D, Sham (100.000 ± 2.345), 7D, SNL (106.399 ± 1.069), 14D, Sham (100.000 ± 3.739), 14D, SNL (110.275 ± 3.868)<br>Two-way ANOVA with Sidak's multiple comparisons test<br>Interaction F (2, 19) = 1.531, p = 0.2418; Time F (2, 19) = 1.870, p = 0.1814; Group F (1.19) = 25.98, p < 0.0001
S2a	Sham Con (0.9393 ± 0.9571), Sham Ipsi (4.410 ± 0.8494), SNL Con (4.895 ± 0.7101), SNL Ipsi (7.072 ± 0.6682)<br>Brown-Forsythe one-way ANOVA with Dunnett T3 multiple comparison test, F (3.000, 231.5) = 8.784, p < 0.0001
S2b	Sham Con (15.12 ± 0.5044), Sham Ipsi (17.24 ± 1.203), SNL Con (13.72 ± 0.6244), SNL Ipsi (14.80 ± 0.5083)<br>Brown-Forsythe one-way ANOVA with Dunnett T3 multiple comparison test, F (3.000, 130.1) = 3.116, p = 0.0285
S2d	Tonic GABA: Sham (1.835 ± 0.6414), SNL (7.314 ± 1.766), SNL+MAOBi (1.420 ± 0.3516)<br>One-way ANOVA with Tukey's multiple comparison test, F (2, 18) = 8.886, p = 0.0021<br>Tonic Glycine: Sham (0.3384 ± 0.2921), SNL (1.193 ± 0.3440), SNL+MAOBi (0.5462 ± 0.4679)<br>One-way ANOVA with Tukey's multiple comparison test, F (2, 18) = 1.411, p = 0.2695
S3b	Sham<br>Intensity of GABA: Con (250.2 ± 10.90), Ipsi (237.5 ± 6.798), Two-tailed unpaired t-test with Welch's correction, p = 0.3250<br>Area of GFAP: Con (1899 ± 204.3), Ipsi (1763 ± 164.7), Two-tailed unpaired t-test with Welch's correction, p = 0.6044<br>SNL<br>Intensity of GABA: Con (358.8 ± 12.78), Ipsi (407.2 ± 10.80), Two-tailed unpaired t-test, p = 0.0043<br>Area of GFAP: Con (2385 ± 257.2), Ipsi (1760 ± 188.4), Two-tailed unpaired t-test, p = 0.0479<br>SNL+MAOBi<br>Intensity of GABA: Con (337.1 ± 12.75), Ipsi (198.3 ± 5.208), Two-tailed unpaired t-test with Welch's correction, p < 0.0001<br>Area of GFAP: Con (2052 ± 215.4), Ipsi (2745 ± 375.9), Two-tailed unpaired t-test with Welch's correction, p = 0.1132
S3d	Sham<br>Con (299.6 ± 22.46), Ipsi (338.9 ± 28.31), Two-tailed unpaired t-test, p = 0.2788<br>SNL<br>Con (291.6 ± 21.76), Ipsi (425.2 ± 36.86), Two-tailed unpaired t-test with Welch's correction, p = 0.0028<br>SNL+MAOBi<br>Con (364.4 ± 21.45), Ipsi (421.9 ± 30.50), Two-tailed unpaired t-test with Welch's correction, p = 0.1279

S4c	Con (1020 ± 35.65), SNL (896 ± 33.83)<br>Unpaired t-test, p = 0.0003
S5a	Sham<br>PO1 (99.82 ± 0.5004), PO4 (103.2 ± 1.754). PO14 (101.1 ± 0.7776)<br>Repeated measure one-way ANOVA with Tukey, F (2, 18) = 1.931, p = 0.1738<br>SNL<br>PO1 (100.4 ± 0.6510), PO4 (107.3 ± 0.6528), PO14 (108.4 ± 1.128)<br>Repeated measure one-way ANOVA adjusted by Geisser-Greenhouse correction with Tukey F (1.594, 12.75) = 27.41, p < 0.001<br>SNL+MAOBi,<br>PO1 (99.65 ± 0.8164), PO4 (108.0 ± 1.298), PO14 (103.4 ± 0.9506)<br>repeated measure one-way ANOVA adjusted by Geisser-Greenhouse correction with Tukey F (1.225, 11.29) = 15.61, p = 0.0014
S5b	Sham<br>PO1 (99.84 ± 0.5003), PO4 (99.93 ± 0.4639), PO14 (99.15 ± 0.5293)<br>Repeated measure one-way ANOVA with Tukey, F (2, 18) = 1.398, p = 0.2726<br>SNL<br>PO1 (99.98 ± 0.55726), PO4 (99.28 ± 0.8186), PO14 (98.26 ± 1.387)<br>Repeated measure one-way ANOVA with Tukey F (2, 24) = 0.7742, p = 0.4722<br>SNL+MAOBi,<br>PO1 (99.05 ± 0.5657), PO4 (98.67 ± 0.5915), PO14 (99.38 ± 0.6003)<br>repeated measure one-way ANOVA with Tukey, F (2, 18) = 0.3045, p = 0.7412
S5c	Sham<br>PO1 (111.2 ± 0.6099), PO4 (110.7 ± 0.7190), PO14 (111.5 ± 0.5908)<br>Repeated measure one-way ANOVA with Tukey, F (2, 18) = 0.4625, p = 0.6370<br>SNL<br>PO1 (112.2 ± 0.5320), PO4 (115.0 ± 0.7658), PO14 (116.6 ± 0.8768)<br>Repeated measure one-way ANOVA adjusted by Geisser-Greenhouse correction with Tukey F (1.613, 12.90) = 17.70, p < 0.001<br>SNL+MAOBi<br>PO1 (111.7 ± 0.5613), PO4 (115.3 ± 1.294), PO14 (112.7 ± 1.333)<br>Repeated measure one-way ANOVA adjusted by Geisser-Greenhouse correction with Tukey, F (1.872, 16.85) = 4.119, p = 0.0371
S5d	Sham<br>PO1 (111.1 ± 0.5055), PO4 (111.7 ± 0.7139), PO14 (112.2 ± 0.8727)<br>Repeated measure one-way ANOVA with Tukey, F (2, 18) = 0.1768, p = 0.8394<br>SNL<br>PO1 (111.9 ± 0.8047), PO4 (111.1 ± 1.317), PO14 (112.5 ± 0.7952)<br>Repeated measure one-way ANOVA adjusted by Geisser-Greenhouse correction with Tukey F (2, 16) = 0.3749, p = 0.6932<br>SNL+MAOBi<br>PO1 (112.4 ± 0.9075), PO4 (112.2 ± 0.8082), PO14 (112.2 ± 1.160)<br>Repeated measure one-way ANOVA adjusted by Geisser-Greenhouse correction with Tukey F (2, 18) = 0.006218, p = 0.9983
S5e	Sham (111.2 ± 0.2326), SNL (114.6 ± 1.290), SNL+MAOBi (113.2 ± 1.071)<br>Repeated measure one-way ANOVA with Tukey. Interaction, F (4, 52) = 4.761, p = 0.0024. Time, F (2, 52) = 7.446, p = 0.0014. Group, F (2, 26) = 7.190, p = 0.0033. Subject, F (26, 52) = 2.472, p = 0.0027).
S5f	Sham (112.0 ± 0.1509), SNL (111.8 ± 0.3794), SNL+MAOBi (112.3 ± 0.049)<br>Repeated measure one-way ANOVA with Tukey. Interaction, F (4, 52) = 0.1165, p = 0.9761. Time, F (1.604, 41.70) = 0.3292, p = 0.6736. Group, F (2, 26) = 0.2308, p = 0.7955. Subject, F (26, 52) = 0.7173, p = 0.8197).
